# Multilayer elastic compression for the treatment of a 30-year venous ulcer^☆^^☆☆^

**DOI:** 10.1016/j.abd.2019.08.025

**Published:** 2020-02-17

**Authors:** Bruna Cristina Velozo, Raquel Colenci, Luciana Patrícia Fernandes Abbade

**Affiliations:** a Department of Nursing, Faculdade de Medicina de Botucatu, Universidade Estadual Paulista Júlio de, Botucatu, SP, Brazil; b Department of Dermatology and Radiotherapy, Faculdade de Medicina de Botucatu, Universidade Estadual Paulista, Botucatu, SP, Brazil

Dear Editor,

Venous Ulcers (VU) are a significant burden for both patients and the health system. Their prevalence and complexity are expected to increase over the years.^1^

The gold standard of treatment for VU, i.e., with a high level of evidence based on systematic reviews, is multilayer elastic compression (MEC).^2^

Although MEC bandages are increasingly used in practice, they remain neglected in many international consensus documents and guidelines related to compression. Added to this, this therapy is not available in the Brazilian Unified Health System, hampering access of the treatment to all patients.^1^

A clinical case with use of MEC in a 73 years old female patient, with a history of post-thrombotic VU of the left lower limb (LLL) for more than 30 years, is reported. Throughout this period several forms of clinical treatments were followed: debridement, local dressings (activated charcoal, hydrofiber silver dressing, hydrogels) and compressive treatment with Unna’s boot and elastic band, without healing of the ulcer.

At the initial examination, an ulcer in the posterior and lateral regions of the LLL was observed with 15 × 8 cm, wound bed with a fibrotic and devitalized tissue, without signs of colonization, edge not adhered to the bed (Fig. 1). The ulcer presented serous exudation and local pain with moderate intensity. Palpable distal pulses 4+/4+. MEC was initiated (Fig. 2) with exchange of dressings twice a week for the first three weeks due to high exudation and saturation of the dressing and, thereafter, once a week. On the first 20 days, the VU became shallower, with partially vitalized bed and edges adhered to the wound bed, making possible the spacing of the dressing changes. In a three-month period, there was a significant improvement in the quality of the ulcer wound bed and a decrease in the ulcerated area until completes healing (Fig. 3). High compression elastic stockings were prescribed to prevent relapse.

**Figure 1:**
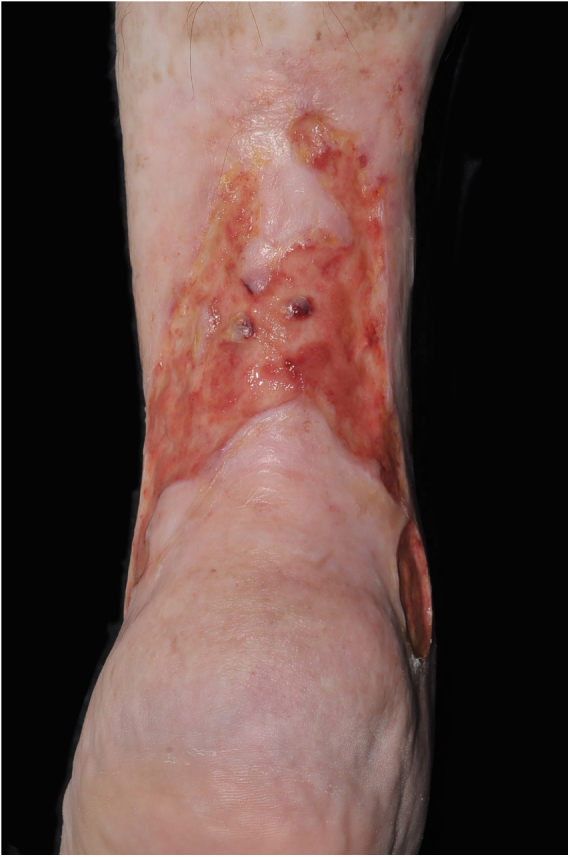
Venous ulcer in the posterior and lateral region of the left lower limb without healing for over 30 years.

**Figure 2:**
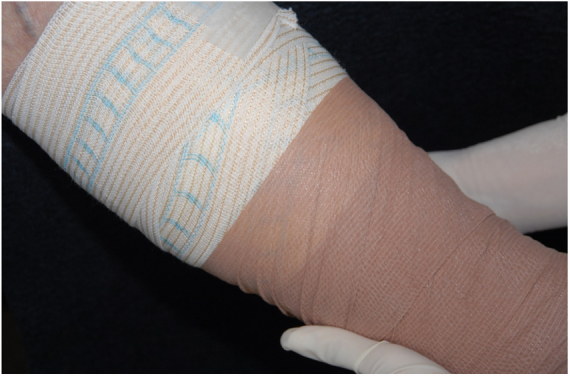
Multilayer elastic compression, performed with a 3 layer system for three months.

**Figure 3:**
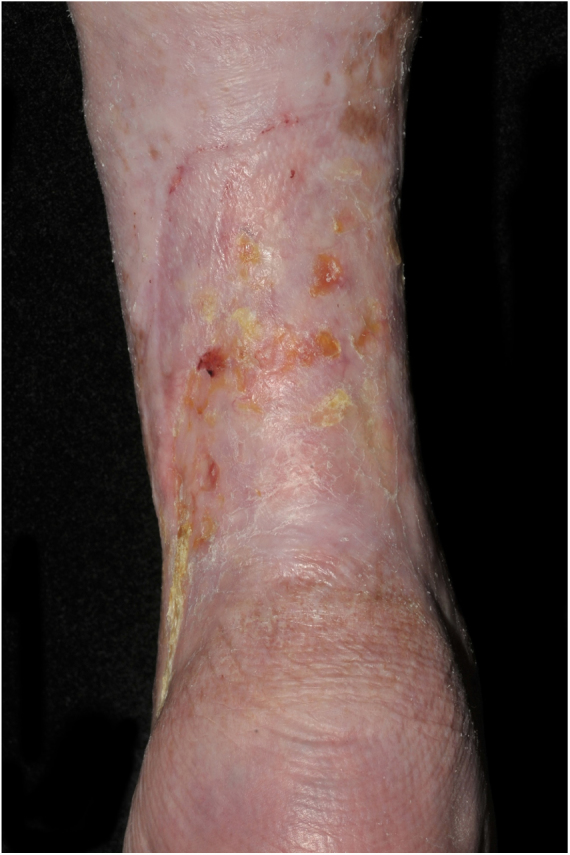
Healed venous ulcer, featuring only some exulcerated and hyperkeratotic areas.

According to a systematic review on compression therapy for VU, compression increases the healing rate. The multilayer system is more effective than the traditional ones, while high compression is more effective than low compression.^3^

There are two basic forms of compressive treatment for VU of the limbs: inelastic (Unna's boot) and elastic (bands, elastic stockings and multilayer systems).

MEC bandages usually have a protective orthopedic wool padding, and may also include a crepe retention layer, elastic compression bandage and cohesive elastic bandage to prevent slipping and keep the system in high compression during usage, becoming effective, because acts both in macro and microcirculation.^4^

The multilayer therapy is composed of 2–4 layers. The following brands are available in Brazil: UrgoK2®, Coban 2®, Jobst® (2 layers), Dyna-flex® (3 layers), Curatec K4® and Profore® by Smith & Nephew® (4 layers). The number of layers is not as essential as the ideal combination of different materials to maintain sustained compression.^3^

A correct diagnosis and exclusion of peripheral arterial disease is important for the indication of MEC. The use of this therapy in patients with absence of distal pulses or ankle-arm index < 0.9 is contraindicated.^2^

This system should remain on the limb for a maximum of seven days, having the advantage of the maintenance of sustained high compression around the ankle throughout this time. The compression should be from 35 to 40 mmHg and gradually lower in the region below the knee (17 mmHg). The patient must be stimulated to ambulate in order to achieve compression's benefits.

MEC bandages should be applied by trained health professionals (physicians and nurses), because their effectiveness may be influenced by the application technique.^1^, ^4^ The unit cost is still high, but the cost/benefit ratio is greater in relation to common dressings and other less effective therapies.

The advantages of MECs are related to the patient's comfort, tolerability and quality of life.^1^ There have been reports on improvement of pain, easiness in performing daily activities and lesser discomfort during the day and during sleep.^5^

The MECs have demonstrated significant effectiveness in healing and comfort to the patient, where our clinical case study demonstrated effectiveness in its use, healing a VU with more than 30 years. This corroborates the systematic reviews regarding the indication of this method, favoring a faster healing with good cost–benefit ratio.

## Financial support

None declared.

## Authors’ contributions

Bruna Cristina Velozo: Elaboration and writing of the manuscript; obtaining, analysis, and interpretation of the data; critical review of the literature.

Raquel Colenci: Effective participation in research orientation; intellectual participation in the propaedeutic and/or therapeutic conduct of the studied cases.

Luciana Patrícia Fernandes Abbade: Approval of the final version of the manuscript; conception and planning of the study; elaboration and writing of the manuscript; effective participation in research orientation; intellectual participation in the propaedeutic and/or therapeutic conduct of the studied cases; critical review of the literature; critical review of the manuscript.

## Conflicts of interest

None declared.
